# *ntrC* Contributes to Nitrogen Utilization, Stress Tolerance, and Virulence in *Acidovorax citrulli*

**DOI:** 10.3390/microorganisms11030767

**Published:** 2023-03-16

**Authors:** Dehua Liu, Mei Zhao, Pei Qiao, Zhanhong Li, Gong Chen, Wei Guan, Qingrong Bai, Ron Walcott, Yuwen Yang, Tingchang Zhao

**Affiliations:** 1 College of Plant Protection, Jilin Agricultural University, Changchun 130118, China; 2 State Key Laboratory for Biology of Plant Diseases and Insect Pests, Institute of Plant Protection, Chinese Academy of Agricultural Sciences, Beijing 100193, China; 3 Department of Plant Pathology, College of Plant Protection, China Agricultural University, Beijing 100193, China; 4 Department of Plant Pathology, University of Georgia, Athens, GA 30602, USA; 5 National Nanfan Research Institute (Sanya), Chinese Academy of Agricultural Sciences, Sanya 572024, China

**Keywords:** *Acidovorax citrulli*, *ntrC*, virulence, nitrogen, nitrate, stress

## Abstract

Bacterial fruit blotch (BFB), caused by *Acidovorax citrulli*, severely damages watermelon, melon, and other cucurbit crops worldwide. Nitrogen, one of the most important limiting elements in the environment, is necessary for the growth and reproduction of bacteria. As a nitrogen-regulating gene, *ntrC* plays an important role in maintaining bacterial nitrogen utilization and biological nitrogen fixation. However, the role of *ntrC* has not been determined for *A. citrulli*. In this study, we constructed a *ntrC* deletion mutant and a corresponding complementary strain in the background of the *A. citrulli* wild-type strain, Aac5. Through phenotype assays and qRT-PCR analysis, we investigated the role of *ntrC* in *A. citrulli* in nitrogen utilization, stress tolerance, and virulence against watermelon seedlings. Our results showed that the *A. citrulli* Aac5 *ntrC* deletion mutant lost the ability to utilize nitrate. The *ntrC* mutant strain also exhibited significantly decreased virulence, in vitro growth, in vivo colonization ability, swimming motility, and twitching motility. In contrast, it displayed significantly enhanced biofilm formation and tolerance to stress induced by oxygen, high salt, and copper ions. The qRT-PCR results showed that the nitrate utilization gene *nasS*; the Type III secretion system-related genes *hrpE, hrpX*, and *hrcJ*; and the pili-related gene *pilA* were significantly downregulated in the *ntrC* deletion mutant. The nitrate utilization gene *nasT*, and the flagellum-related genes *flhD, flhC, fliA*, and *fliC* were significantly upregulated in the *ntrC* deletion mutant. The expression levels of *ntrC* gene in the MMX-q and XVM2 media were significantly higher than in the KB medium. These results suggest that the *ntrC* gene plays a pivotal role in the nitrogen utilization, stress tolerance, and virulence of *A. citrulli*.

## 1. Introduction

Bacterial fruit blotch (BFB), caused by *Acidovorax citrulli*, is a seed-borne bacterial disease that can infect and cause significant economic losses to cucurbit crops, including watermelon (*Citrullus lanatus*) and melon (*Cucumis melo*). The disease was first reported in Georgia, USA, in 1965 and has been reported in many countries [1]. *Acidovorax citrulli* uses a variety of virulence factors, such as the Type III secretion system (T3SS), the Type VI secretion system (T6SS), Type IV pili (T4P), polar flagella, quorum sensing, and biofilms [2,3,4,5,6,7,8,9,10] to induce disease. *Acidovorax citrulli* strains can be divided into two major groups (I and II) [11,12]. Natural field experiments showed that *A. citrulli* groups (I and II) displayed a preferential association for different cucurbit hosts [13,14]. Despite efforts to develop strategies to mitigate the losses caused by BFB, the current management strategies are not effective, and outbreaks continue to occur sporadically, with economic consequences [15].

As one of the basic elements of life, nitrogen is necessary for the growth of bacteria [16]. Bacteria can utilize nitrogen-containing compounds such as ammonium salts and nitrates as nitrogen sources [16,17]. The utilization of nitrate and nitrite by bacteria mainly depends on the nitrate and nitrite assimilation reductase system, which can convert nitrate and nitrite in the environment into ammonium. This ammonium can then be used in the glutamate dehydrogenase reaction [16]. The utilization of ammonium salts from the environment by bacteria is mainly through the diffusion of NH_4_^+^ across the cell membrane. The membrane protein AmtB has a high affinity for NH_4_^+^ and plays an important role in the absorption of NH_4_^+^ when it is limiting [16]. The two-component system, NtrBC, plays a key role in bacterial regulation of nitrogen. When nitrogen is limiting, the amount of glutamine inside the cells is reduced compared with 2-ketoglutarate, which causes uridylation of PII, the inhibition of NtrB phosphatase activity, and the activation of NtrC [17]. After being phosphorylated, NtrC, together with RNA polymerase and σ^54^, activates the expression of different genes [17]. The deletion of the response regulator *ntrC* gene in *Rhizobium leguminosarum*, *Sinorhizobium fredii*, *Pseudomonas fluorescens,* and *Agrobacterium* sp. had different effects on the utilization of nitrogen sources such as nitrate, ammonium salts, and urea [18,19,20,21].

In addition to regulating nitrogen, NtrC is also involved in many biological processes in bacteria, such as stress tolerance, extracellular polymer synthesis, and biofilm formation [22,23,24,25]. The deletion of *ntrC* reduced in vitro growth but enhanced oxygen-related stress tolerance in *P. putida* [22]. The deletion of *ntrC* resulted in significant changes in the tolerance of *Aeromonas hydrophila* to osmotic stress, heavy metal ions, oxidation, and different antibiotic stresses [23]. Interestingly, swimming motility and virulence decreased in the *P. aeruginosa ntrB*-*ntrC* deletion mutant [24]. The biofilm production of a *Vibrio cholerae ntrC* mutant was significantly higher than that of the wild-type strain [25]. Despite these observations, the role of *ntrC* in the virulence of plant pathogenic bacteria is not known. Therefore, in this study, we constructed *an ntrC* gene deletion mutant in *A. citrulli* and used it to investigate the effect of *ntrC* on virulence, nitrogen utilization, and stress tolerance. We also analyzed the regulatory network that *ntrC* participates in.

## 2. Materials and Methods

### 2.1. Plant Materials, Bacterial Strains, and Plasmids

The watermelon cultivar ‘Ruixin’ (provided by China Vegetable Seed Technology Corporation, Beijing, China) was used in seed-to-seedling transmission assays as previously described [2,9,13]. The bacterial strains and plasmids used in this study are shown in Table S1.

The wild-type (Aac5-WT), mutant (Aac5Δ*ntrC*), and complementary (Aac5Δ*ntrC*comp) strains of *A. citrulli* were cultivated at 28 °C in King’s B (KB) medium [10], and *Escherichia coli* was cultivated at 37 °C in a lysogeny broth (LB) medium [10]. The working concentrations of antibiotics used in this study were 100 μg·mL^−1^ ampicillin (Amp) and 50 μg·mL^−1^ kanamycin (Kan).

### 2.2. Construction of the A. citrulli ntrC Mutant and Its Complementary Strain

The primer pair *ntrC*-F/R was designed on the basis of the *ntrC* gene (*Aave_1445*) in the AAC00-1 genome (GenBank accession number: CP000512.1), and the primer pairs, *ntrC*-1F/1R and *ntrC*-2F/2R, were designed on the basis of the upstream and downstream flanking sequences of the *ntrC* gene (Table S2). The nitrogen regulatory gene *ntr*C was deleted using the double homologous recombination approach, as previously described [2]. Briefly, the sequences 592 bp upstream and 532 bp downstream of the *ntrC* gene were amplified from the genomic DNA of Aac5-WT using KOD-Plus-Neo (TOYOBO, Osaka, Japan) and the primers *ntrC*-1F/*ntrC*-1R and *ntrC*-2F/*ntrC*-2R. These two flanking sequences were fused by an overlapping polymerase chain reaction (PCR) and then ligated into the suicide vector pK18*mobsacB* using the ClonExpress II One Step Cloning Kit (Vazyme, Shanghai, China) to generate pK18*mobsacB-ntrC,* which was then transformed into competent *E. coli* DH5α [26]. The vector pK18*mobsacB-ntrC* was confirmed by sequencing. The DNA sequencing in this study was performed by Liuhe BGI Co., Ltd. (Beijing, China). Then the vector pK18*mobsacB-ntrC* was introduced into Aac5-WT by triparental hybridization, and the individual crossover colonies were screened on the basis of their Amp and Kan resistance. The mutant was screened on an M9 agar medium with sucrose [27,28]. The mutant strain Aac5Δ*ntrC* was verified by PCR and DNA sequencing. To construct the complementary strain, the *ntrC* gene sequence with its native promoter sequence was ligated into pBBR1MCS-2 to generate pBBR1MCS-*ntrC*. Then the pBBR1MCS-*ntrC* was introduced into the mutant Aac5Δ*ntrC* by triparental hybridization and verified by PCR and DNA sequencing. To eliminate the effect of pBBR1MCS-2, the empty vector pBBR1MCS-2 was introduced into Aac5-WT and Aac5Δ*ntrC* by triparental hybridization and verified by PCR and DNA sequencing.

### 2.3. Determination of the Nitrogen Utilization Capacity and In Vitro Growth Ability

To assess the ability of bacterial strains to use different nitrogen sources, (NH_4_)_2_SO_4_ (10 mmol·L^−1^), KNO_3_ (20 mmol L^−1^), and CH_4_N_2_O (10 mmol·L^−1^) were added separately in the MMX basic medium [29]. Overnight cultures of Aac5, Aac5Δ*ntrC*, and Aac5Δ*ntrC*comp were adjusted to OD_600_ = 0.6 after resuspension with sterilized distilled water, and the bacterial suspensions (10 μL) were added to 2 mL centrifuge tubes with 1 mL of the medium (MMX-(NH_4_)_2_SO_4_, MMX-KNO_3_, and MMX-CH_4_N_2_O) (three replicates). The suspensions were cultured at 28 °C under shaking at 220 revolutions·min^−1^ for 96 h, and the OD_600_ values were determined using a spectrophotometer (Biochrom, Cambridge, UK). The experiment was conducted three times.

The bacterial suspensions of Aac5, Aac5Δ*ntrC*, and Aac5Δ*ntrC*comp were resuspended with MMX-KNO_3_ or KB medium, adjusted to OD_600_ = 0.3, and diluted 100-fold using the MMX-KNO_3_ or KB medium. Then the diluted bacterial suspensions (200 μL) were added to 100-well polystyrene plates. The plates were incubated at 28 °C with continuous shaking, and the OD_600_ values was measured every 2 h for 96 h (Bioscreen C° PRO, Finland) [6]. Each treatment was replicated four times, and the experiment was conducted at least three times.

### 2.4. Determination of Tolerance to Stress

#### 2.4.1. Hydrogen Peroxide Sensitivity Assay

Bacterial suspensions of Aac5, Aac5Δ*ntrC*, and Aac5Δ*ntrC*comp were generated in sterilized water and adjusted to a final concentration of 3 × 10^8^ colony-forming units (CFU)·mL^−1^ (OD_600_ = 0.3). Bacterial suspensions (2.5 mL) were mixed evenly with 50 mL of melted KB agar medium. After the medium had solidified, 5 μL of a 3% H_2_O_2_ solution was placed on the plate (three replicates). The diameters of the zones of inhibition were measured 2 days after incubation at 28 °C. The experiment was conducted three times.

#### 2.4.2. Sodium Chloride and Copper Sulphate Sensitivity Assays

Bacterial suspensions of Aac5, Aac5Δ*ntrC*, and Aac5Δ*ntrC*comp were adjusted to OD_600_ = 0.3 and centrifuged, and the pellets were resuspended in KB media containing 4% NaCl or KB media containing 4 mmol CuSO_4_·5H_2_O, and incubated at 28 °C with shaking at 220 r·min^−1^ for 20 h. Aliquots were serially diluted 10-fold and were plated on KB agar media (containing 1.5% agar) supplemented with appropriate concentrations of Amp and Kan. Colonies were counted 48 h after incubation at 28 °C. Each treatment was replicated 3 times and the experiment was conducted 3 times.

### 2.5. Virulence Assays

#### 2.5.1. Spray Inoculation Assay

Overnight cultures of Aac5, Aac5Δ*ntrC*, and Aac5Δ*ntrC*comp were adjusted to OD_600_ = 0.3. For each treatment, four watermelon seedlings (4 weeks old) were sprayed with 10 mL of the bacterial suspension or water (negative control) and then bagged to maintain high relative humidity. The inoculated seedlings were placed in a growth chamber under the conditions of light at 28 °C for 16 h and darkness at 22 °C for 8 h, and a relative humidity of 80%. The disease index was evaluated and calculated at 5 days post-inoculation (dpi) [30]. The experiment was conducted three times.

#### 2.5.2. Seed-to-Seedling Transmission Assay

Overnight cultures of Aac5, Aac5Δ*ntrC*, and Aac5Δ*ntrC*comp were adjusted to OD_600_ = 0.3. Then watermelon seeds (three replicates per treatment, 10 seeds per replicate) were soaked in the bacterial suspension for 1 h. Seeds were soaked in sterilized water as a negative control. Inoculated seeds were then air-dried and planted in nutritive substrates (Guangdahengyi, Beijing, China). The watermelon seedlings were cultured in a growth chamber under the conditions described above. The disease index was evaluated and calculated at 14 days after sowing [31]. The experiment was conducted three times.

### 2.6. In Vivo Growth Ability

The concentrations of the bacterial suspensions of the strains Aac5, Aac5Δ*ntrC*, and Aac5Δ*ntrC*comp were adjusted to 10^6^ CFU·mL^−1^ with sterilized water. Each bacterial suspension was injected into watermelon cotyledons (three replicates per treatment, 15 cotyledons per replicate) with a 1 mL syringe and incubated in a growth chamber under the same incubation conditions as described above. Sterilized water was used as a negative control. At 1, 24, 48, 72, and 96 h post-inoculation (hpi), watermelon cotyledons were selected for observation and photography. One leaf disk (0.8 cm in diameter) for each cotyledon was sampled, and each replicate constituted three cotyledons. Three leaf disks were macerated in 500 μL of sterilized water in a microcentrifuge tube. The macerates were serially diluted 10-fold, and plated on KB agar media with Amp and Kan. Colonies were counted 48 h after incubation at 28 °C [32]. The assays were repeated three times.

### 2.7. Assays of Swimming and Twitching Motility Ability

To assess swimming motility, bacterial suspensions of Aac5, Aac5Δ*ntrC*, and Aac5Δ*ntrC*comp (OD_600_ = 0.3, 3 μL) were gently inoculated onto the surfaces of a 0.3% semisolid medium (tryptone, 0.3 g; yeast extract, 0.3 g; agar, 3 g) [33] and incubated at 28 °C for 48 h. Each colony halo was photographed and its diameter was measured. Every treatment had four replicates and the experiment was conducted three times.

To assess twitching motility, fresh colonies of Aac5, Aac5Δ*ntrC*, and Aac5Δ*ntrC*comp were streaked onto KB agar media. Corrugated tracks around each colony were observed with an IX83 inverted microscope (OLYMPUS, Tokyo, Japan) after 4 days of incubation at 28 °C [5]. The ratio of the halo’s diameter compared with the colony’s diameter was determined. Each treatment was replicated six times and the experiment was conducted three times.

### 2.8. Biofilm Formation Assay

Aac5, Aac5Δ*ntrC*, and Aac5Δ*ntrC*comp liquid cultures suspended in KB broth were adjusted to OD_600_ = 0.3, and 2 mL of each suspension was added to 12-well polystyrene plates with three replicates for each treatment. The plates were incubated at 28 °C for 72 h, and then the liquid was slowly removed with a pipette, washed slowly with sterilized water, and fixed in an oven at 80 °C for 30 min. The wells were stained with 0.1% crystal violet for 50 min and rinsed three times with sterilized water. The plates were air-dried at 37 °C and photographed. Two milliliters of 95% ethanol needed to be added to dissolve the biofilm formed by Aac5, Aac5Δ*ntrC*, and Aac5Δ*ntrC*comp, and the OD_575_ values were measured using a spectrophotometer. The experiment was conducted three times [7].

### 2.9. Determination of the Expression of Nitrate Assimilation- and Virulence-Related Genes 

Aac5 and Aac5Δ*ntrC* were cultured in a KB medium, a T3SS induction XVM2 medium (sucrose, 3.432 g; fructose, 1.801 g; casein hydrolysate, 0.3 g; (NH_4_)_2_SO_4_, 1.33 g; NaCl, 1.17 g; CaCl_2_, 1.11 g; FeSO_4_·7H_2_O, 0.0028 g; MgSO_4_, 0.601 g; KH_2_PO_4_, 0.0217 g; K_2_HPO_4_·3H_2_O, 0.073 g; deionized water, 1000 mL; pH 6.7) [34], and a MMX-q medium (glucose, 5 g; (NH_4_)_2_SO_4_, 0.133 g; KNO_3_, 1.8 g; MgSO_4_·7H_2_O, 0.2 g; K_2_HPO_4_, 4 g; KH_2_PO_4_, 6 g; trisodium citrate, 1 g; deionized water, 1000 mL). The total RNA was extracted from each strain using a bacterial total RNA extraction kit (Yeasen, Shanghai, China), and the RNA was reverse-transcribed into cDNA using a FastQuent RT Kit (TianGen, Beijing, China). *rpoB* was selected as an internal reference gene; *nasS* and *nasT* were selected as the key genes for nitrate assimilation; *hrpG*, *hrpE*, *hrpX*, and *hrcJ* were selected as the key genes of T3SS; *flhD*, *flhC*, *fliA*, *fliC*, and *fliM* were selected as flagellum-related genes; and *pilA* and *pilN* were selected as pili-related genes. Gene expression levels were determined by quantitative real-time PCR (qRT-PCR). For testing the expression of *nasS* and *nasT*, strains were induced in an MMX-q medium. For testing the expression of *hrpG*, *hrpE*, *hrpX*, and *hrcJ*, strains were induced in an XVM2 medium. For testing the expression of *flhD*, *flhC*, *fliA*, *fliC*, *fliM*, *pilA*, and *pilN*, strains were induced in a KB medium. The primers used in this assay are shown in Table S2. The average value of the expression of related genes in Aac5 was set to 1, the corresponding Ct values were recorded, and the relative gene expression levels were calculated [35,36,37]. These experiments were performed three times independently.

### 2.10. Determination of the Expression of the ntrC Gene in Different Media

The expression of *ntrC* by Aac5 in the KB medium, the T3SS induction XVM2 medium, and the MMX-q medium was determined as described above, and *rpoB* was used as an internal reference gene. Each treatment had three replicates. This experiment was performed three times independently.

### 2.11. Data Analysis

The experimental data were recorded and calculated using Excel (Microsoft, Redmond, WA, USA), and graphs were plotted using GraphPad Prism 7 (GraphPad, San Diego, CA, USA). For statistical analysis, one-way analysis of variance (ANOVA) was conducted using GraphPad Prism 7 (with 95% confidence intervals).

## 3. Results

### 3.1. Confirmation of the Mutant and Complementary Strains

The full length of the *ntrC* gene is 1650 bp long and is located at genomic nucleotide positions 1592112 to 1593761 of *A*. *citrulli* Group II strain AAC00-1. Aac5Δ*ntrC* was verified by PCR using the *ntrC*-specific verification primer Δ*ntrC*-F/Δ*ntrC*-R, the *A. citrulli*-specific primer WFB1/WFB2, and the pBBR1MCS-2 plasmid detection primer Kan-F/Kan-R (Table S2), and by sequencing. The complementary strain Aac5Δ*ntrC*comp showed resistance to Kan, and was also verified by PCR and sequencing.

### 3.2. Inactivation of ntrC Affects A. citrulli’s Ability to Assimilate Nitrogen and Grow In Vitro

**Figure 1:**
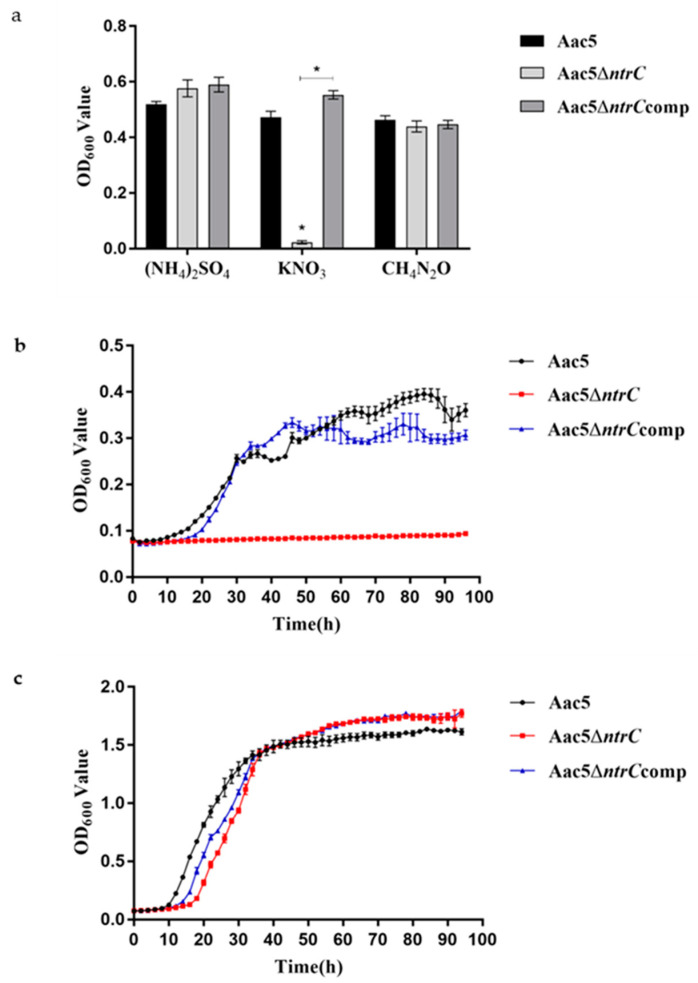
Inactivation of *ntrC* affected nitrogen assimilation and in vitro growth in *Acidovorax citrulli*. (**a**) The OD_600_ value of the strains Aac5, Aac5Δ*ntrC*, and Aac5Δ*ntrC*comp after 96 h of incubation in a basic MMX medium. Nitrogen sources (NH_4_)_2_SO_4_ (10 mmol·L^−1^), KNO_3_ (20 mmol L^−1^), and CH_4_N_2_O (10 mmol·L^−1^) were added separately to the basic MMX medium. Each treatment had three replicates, and the experiment was conducted three times. Asterisks indicate significant differences; error bars represent standard errors of the means (*p* < 0.05, one-way ANOVA test). (**b**) Growth curve of the strains Aac5, Aac5Δ*ntrC*, and Aac5Δ*ntrC*comp in the MMX-KNO_3_ medium at 28 °C for 96 h. The OD_600_ values were measured every 2 hours. Each treatment had four replicates, and the experiment was conducted three times. (**c**) Growth curves of Aac5, Aac5Δ*ntrC*, and Aac5Δ*ntrC*comp in the KB medium at 28 °C for 96 h. Each treatment had four replicates, and the experiment was conducted three times.

Aac5Δ*ntrC* was unable to utilize KNO_3_ as a sole nitrogen source, but could utilize (NH_4_)_2_SO_4_ and CH_4_N_2_O as a sole nitrogen source. In contrast, Aac5 and Aac5Δ*ntrC*comp cells grew on KNO_3_, (NH_4_)_2_SO_4_, and CH_4_N_2_O (Figure 1a). We further examined the in vitro growth dynamics of Aac5, Aac5Δ*ntrC*, and Aac5Δ*ntrC*comp on KNO_3_ as the sole nitrogen source. During the 96 h incubation period, both Aac5 and Aac5Δ*ntrC*comp reached a plateau (OD_600_ = 0.3) after a logarithmic growth period, while the OD_600_ value of Aac5Δ*ntrC* remained unchanged from 0 h to 96 h (Figure 1b). The results showed that *ntrC* was critical for *A. citrulli* to utilize nitrate. In the KB medium, Aac5Δ*ntrC* entered the logarithmic growth phase about 8 h later than Aac5. However, after entering the stationary phase, Aac5Δ*ntrC* reached a higher OD_600_ than Aac5 (Figure 1c). Additionally, the time when the complementary strain Aac5Δ*ntrC*comp entered the logarithmic phase was between those of Aac5 and Aac5Δ*ntrC*, but the growth in the stationary phase was the same as that of Aac5Δ*ntrC* and higher than that of Aac5 (Figure 1c).

### 3.3. ntrC Deletion Affects the Stress Tolerance of Aac5

**Figure 2:**
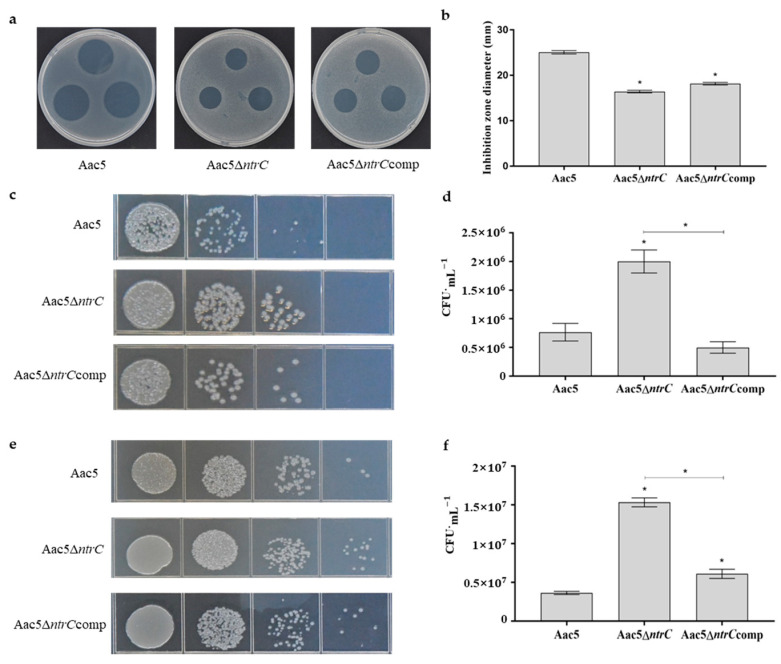
Effect of *ntrC* on tolerance to oxidative stress, high salt stress, and Cu^2+^ stress in *A. citrulli*. (**a**) The ability of Aac5, Aac5Δ*ntrC*, and Aac5Δ*ntrC*comp to tolerate oxidative stress was determined using the inhibitory halo method. After the medium had solidified, 5 μL of a 3% H_2_O_2_ solution was placed on the plate. The plates were photographed 2 days after plating. Each treatment included three replicates and the experiment was conducted three times. (**b**) The average diameter of the inhibition zone of Aac5, Aac5Δ*ntrC*, and Aac5Δ*ntrC*comp measured after 2 days of incubation. (**c**,**d**) The viable bacterial counts of Aac5, Aac5Δ*ntrC*, and Aac5Δ*ntrC*comp after 20 h of culture in a KB medium containing 4% sodium chloride. (**e**,**f**) The viable bacterial counts of Aac5, Aac5Δ*ntrC*, and Aac5Δ*ntrC*comp after 20 h of culture in a KB medium containing 4 mmol CuSO_4_·5H_2_O. Each treatment had three replicates, and the assays were repeated three times. Asterisks indicate significant differences; error bars represent the standard errors of the means (*p* < 0.05, one-way ANOVA test).

The average diameters of the inhibition zones of Aac5, Aac5Δ*ntrC*, and Aac5Δ*ntrC*comp were 25.08 mm, 16.42 mm, and 18.17 mm, respectively, on KB agar. The diameter of Aac5Δ*ntrC* was significantly smaller than that of Aac5 (*p* < 0.05). However, the diameter of the inhibition zone of the complementary strain Aac5Δ*ntrC*comp was larger than that of Aac5Δ*ntrC* but less than that of Aac5 (Figure 2b). The results showed that deletion of the *ntrC* gene enhanced the ability of *A. citrulli* to tolerate oxygen stress.

The tolerance of Aac5 and its derived strains to high salt stress was determined using a KB medium containing 4% sodium chloride. After incubation under high salt stress for 20 h, the average surviving population of Aac5, Aac5Δ*ntrC*, and Aac5Δ*ntrC*comp was 7.67 × 10^5^ CFU·mL^−1^, 2.0 × 10^6^ CFU·mL^−1^, and 5.0 × 10^5^ CFU·mL^−1^, respectively. The surviving population of Aac5Δ*ntrC* was significantly higher than that of Aac5 (*p* < 0.05), while that of Aac5Δ*ntrC*comp could be restored to the level of Aac5 (Figure 2d). The results showed that the deletion of *ntrC* enhanced the ability of *A. citrulli* to tolerate high salt stress.

After incubation in a KB medium with 4 mmol CuSO_4_·5H_2_O for 20 h, the average surviving population of Aac5, Aac5Δ*ntrC*, and Aac5Δ*ntrC*comp was 3.63 × 10^6^ CFU·mL^−1^, 1.53 × 10^7^ CFU·mL^−1^, and 6.1 × 10^6^ CFU·mL^−1^, respectively. The surviving population of Aac5Δ*ntrC* was significantly higher than that of Aac5 (*p* < 0.05). The surviving population of Aac5Δ*ntrC*comp was between that of Aac5Δ*ntrC* and Aac5 (Figure 2f). The results showed that deletion of the *ntrC* gene significantly increased tolerance to copper in *A. citrulli*.

### 3.4. ntrC Contributes to the Virulence of A. citrulli Aac5 

To elucidate the role of the *ntrC* gene in the virulence of *A. citrulli*, we carried out watermelon spray-inoculation assays and seed-to-seedling transmission assays.

**Figure 3:**
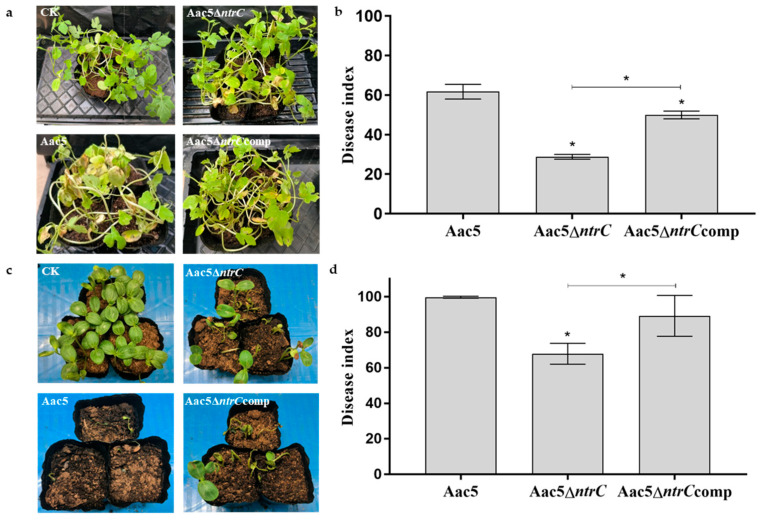
Virulence assay of *Acidovorax citrulli* strains Aac5, Aac5Δ*ntrC*, and Aac5Δ*ntrC*comp on watermelon seedlings. (**a**) Bacterial fruit blotch symptoms on watermelon leaves at 5 days post-inoculation (dpi). CK: negative control inoculated with water. Watermelon seedlings were spray-inoculated with bacterial suspensions (3 × 10^8^ CFU·mL^−1^). Each treatment had four replicates, and the experiment was conducted three times. (**b**) The disease index of watermelon seedlings at 5 dpi. (**c**) Watermelon seeds were soaked in sterilized water (CK), and Aac5, Aac5Δ*ntrC*, and Aac5Δ*ntrC*comp suspensions for 1 h, and photos were taken at 14 dpi. Each treatment had three replicates and the experiment was conducted three times. (**d**) The disease index of watermelon seedlings 14 days after sowing. Asterisks indicate significant differences; the error bars represent the standard errors of the means (*p* < 0.05, one-way ANOVA test).

The disease symptoms on seedlings inoculated with Aac5Δ*ntrC* were less severe than in seedlings inoculated with Aac5 at 5 dpi (Figure 3a). The disease index for watermelon seedlings inoculated with Aac5Δ*ntrC* was 28.80, which was significantly lower than that of watermelon seedlings inoculated with Aac5 (61.77, *p* < 0.05). The disease index of Aac5Δ*ntrC*comp recovered to 49.99 (Figure 3b).

The virulence of each strain was also determined using seed-to-seedling transmission assays. The disease symptoms on seedlings inoculated with Aac5Δ*ntrC* were less severe than those for seedlings inoculated with Aac5 14 days after sowing (Figure 3c). The disease index of watermelon seedlings inoculated with Aac5Δ*ntrC* was 66.20, significantly lower than that of watermelon seedlings inoculated with Aac5 (99.54, *p* < 0.05). The disease index of Aac5Δ*ntrC*comp recovered to 86.11 (Figure 3d).

### 3.5. Deletion of ntrC Reduces the In Vivo Growth of A. citrulli Aac5

**Figure 4:**
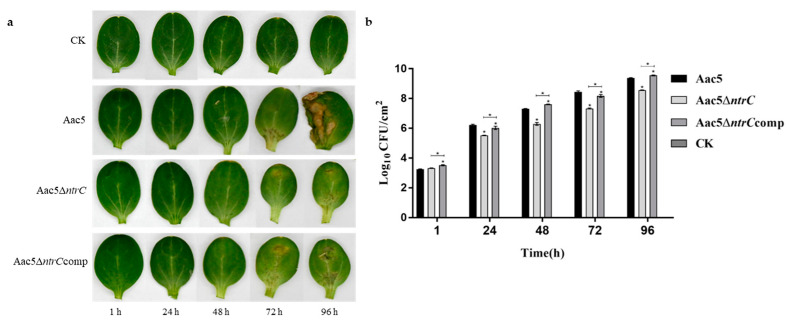
In vivo growth of *Acidovorax citrulli* strains tested in watermelon cotyledons. (**a**) The symptoms of watermelon cotyledons inoculated with Aac5, Aac5Δ*ntrC*, Aac5Δ*ntrC*comp, and sterilized water (CK) at 1, 24, 48, 72, and 96 hpi. Each treatment had three replicates, and the experiment was conducted three times. (**b**) Bacterial population levels in watermelon cotyledons inoculated with the tested strains at 1, 24, 48, 72, and 96 hpi. Error bars represent the standard errors of the means. * indicates significant statistical differences (one-way ANOVA test, *p* < 0.05).

Cotyledons infiltrated with strains Aac5, Aac5Δ*ntrC*, and Aac5Δ*ntrC*comp gradually developed BFB symptoms, but cotyledons treated with Aac5Δ*ntrC* showed only mild symptoms. As expected, cotyledons treated with sterilized water showed no symptoms (Figure 4a). The bacterial population in cotyledons of Aac5Δ*ntrC* was significantly lower than that of Aac5 between 24 hpi and 96 hpi (*p* < 0.05), while the in vivo growth of Aac5Δ*ntrC*comp basically returned to the wild-type levels (Figure 4b).

### 3.6. Deletion of ntrC Impairs Swimming and Twitching Motility in A. citrulli

**Figure 5:**
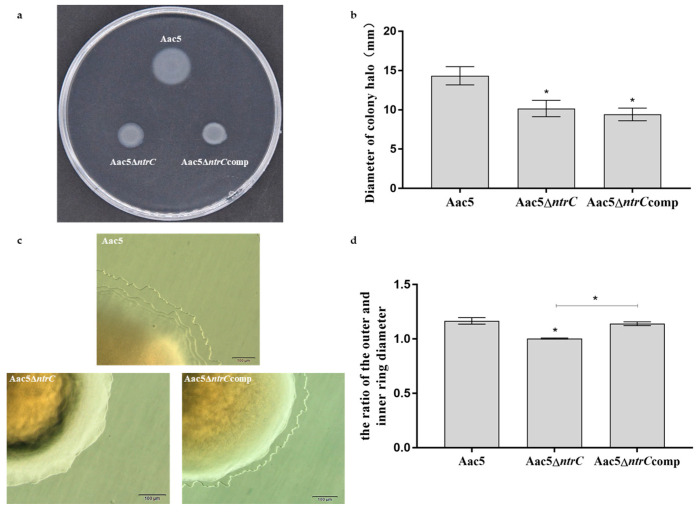
Deletion of *ntrC* impairs swimming and twitching motility in *Acidovorax citrulli*. (**a**) The swimming motility of the tested strains appeared as white halos on a 0.3% agar medium after 48 h of incubation. Each treatment had four replicates, and the experiment was conducted three times. (**b**) The average halo diameter of the strains Aac5, Aac5Δ*ntrC*, and Aac5Δ*ntrC*comp. (**c**) Twitching motility of Aac5, Aac5Δ*ntrC*, and Aac5Δ*ntrC*comp incubated on KB plates for 96 h. Each treatment had six replicates, and the assays were repeated three times. (**d**) The ratio of the outer halo’s diameter to the inner circle’s diameter for Aac5, Aac5Δ*ntrC*, and Aac5Δ*ntrC*comp. Asterisks indicate significant differences; error bars represent the standard errors of the means (*p* < 0.05, one-way ANOVA test).

The average diameters of the Aac5, Aac5Δ*ntrC*, and Aac5Δ*ntrC*comp colonies were 14.33 mm, 10.17 mm, and 9.42 mm, respectively. Compared with Aac5, the swimming motility of Aac5Δ*ntrC* was significantly reduced, and complementation did not restore this ability (Figure 5a,b). The twitching motility of Aac5Δ*ntrC* was also significantly reduced compared with Aac5, while that of Aac5Δ*ntrC*comp was restored to the wild-type levels (Figure 5c,d).

### 3.7. ntrC Contributes to Biofilm Formation in A. citrulli Aac5

**Figure 6:**
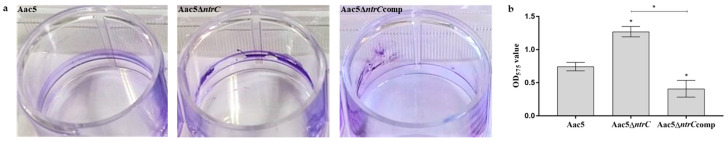
Biofilm formation of the *Acidovorax citrulli* strains Aac5, Aac5Δ*ntrC*, and Aac5Δ*ntrC*comp. Each treatment had three replicates, and the experiment was conducted three times. (**a**) Tested strains formed visible biofilms on the inner wall of the culture wells in a KB medium after 3 days of incubation. (**b**) The formation of a biofilm was quantified by measuring the optical density of the stained biofilm at wavelength of 575 nm. Asterisks indicate significant differences; error bars represent the standard errors of the means (*p* < 0.05, one-way ANOVA test).

We determined the biofilm formation ability of Aac5, Aac5Δ*ntrC*, and Aac5Δ*ntrC*comp by culturing them in a KB medium. Aac5, Aac5Δ*ntrC*, and Aac5Δ*ntrC*comp formed visible biofilms on the inner walls of the 12-well cell culture plates (Figure 6a). The OD_575_ values of Aac5, Aac5Δ*ntrC*, and Aac5Δ*ntrC*comp were 0.74, 1.27, and 0.41, respectively. The OD_575_ value of Aac5Δ*ntrC* was significantly higher than that of Aac5, while the absorbance of the complementary strain was significantly lower than that of Aac5 (*p* < 0.05) (Figure 6b).

### 3.8. Effect of ntrC on the Expression of Select A. citrulli Genes 

**Figure 7:**
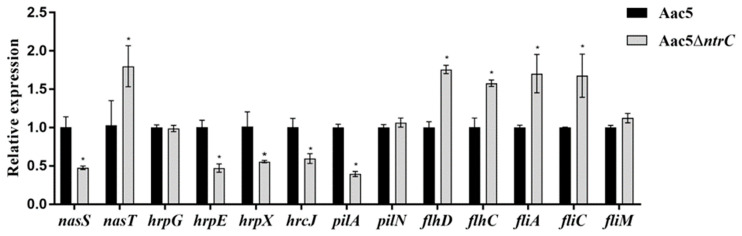
Analysis of the expression of key genes in *Acidovorax citrulli* strains Aac5 and Aac5Δ*ntrC*. *rpoB* was used as an internal reference gene. Each treatment had three replicates, and the experiment was conducted three times. Asterisks indicate significant differences; error bars represent the standard errors of the means (*p* < 0.05, one-way ANOVA test).

The expression of the nitrate utilization-related gene *nasS* was significantly decreased while *nasT* was significantly increased in the MMX-q medium. The expression of the key T3SS genes *hrpE*, *hrpX*, and *hrcJ* was significantly decreased in the XVM2 medium in Aac5Δ*ntrC* compared with Aac5. However, the expression of *hrpG* was not significantly different. When cultured in the KB medium, the expression of the Aac5Δ*ntrC* flagellum-related genes *flhD*, *flhC*, *fliA*, and *fliC* increased significantly in Aac5Δ*ntrC* compared with Aac5, while the pili-related gene *pilA* decreased significantly. The expression levels of *fliM* and *pilN* were not significantly different (Figure 7). Overall, there appeared to be a regulatory relationship of *A. citrulli ntrC* with T3SS-related genes, nitrate utilization-related genes, flagellum-related genes, and pili-related genes.

### 3.9. Expression of the A. citrulli ntrC Gene in Different Media

**Figure 8:**
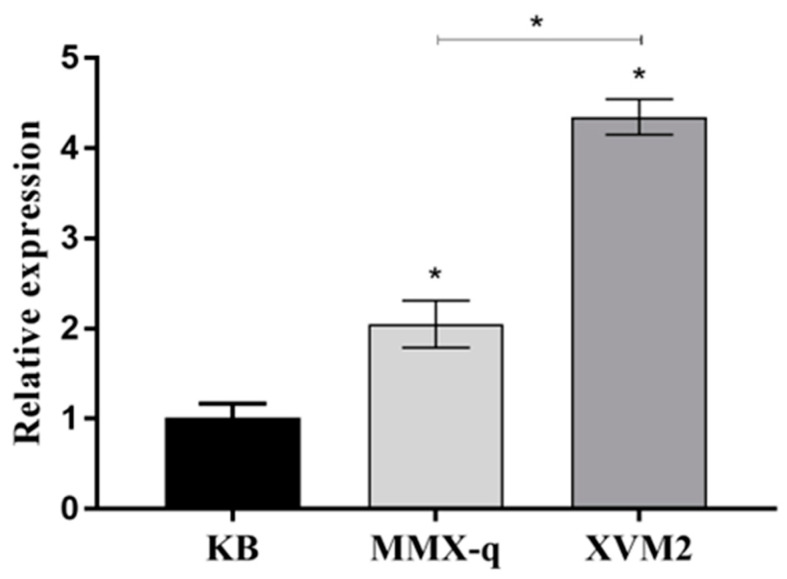
Relative expression of *ntrC* in *Acidovorax citrulli* strain Aac5 in KB, MMX-q, and XVM2 media. qRT-PCR assays were conducted, and *rpoB* was used as an internal reference gene. Each treatment had three replicates, and the experiment was conducted three times. Asterisks indicate significant differences; error bars represent the standard errors of the means (*p* < 0.05, one-way ANOVA test).

The expression levels of Aac5 *ntrC* in the MMX-q and XVM2 media were significantly higher than in the KB medium (*p* < 0.05). The expression of *ntrC* by Aac5 in the XVM2 medium was significantly higher than in the MMX-q medium (*p* < 0.05) (Figure 8).

## 4. Discussion

In this study, we constructed a *ntrC* deletion mutant and a corresponding complemented strain in the background of the *A. citrulli* wild-type strain Aac5. Through phenotype assays and qRT-PCR analysis, we investigated the role of the *ntrC* gene in nitrogen utilization, stress tolerance, and virulence against watermelon seedlings in *A. citrulli*.

We observed that the *ntrC* gene deletion mutant was unable to use potassium nitrate as the sole nitrogen source, but could grow with ammonium sulfate and urea as the sole nitrogen sources. In addition, the expression of the nitrate utilization-related gene *nasS* was significantly downregulated, and the expression of *nasT* was significantly upregulated in Aac5Δ*ntrC*. NasS and NasT comprise the two-component system that regulates bacterial nitrate utilization [38]. We speculate that *ntrC* affects the activation of the *nasS* promoter and further affects the utilization of nitrate, which is consistent with previous studies [38]. The expression levels of *ntrC* by Aac5 in the MMX-q and XVM2 media were significantly higher than in the KB medium, indicating that when nutrients are limiting, the *ntrC* gene is highly expressed to regulate the critical intracellular metabolic pathways. In addition, in the MMX-q medium, deletion of the *ntrC* gene upregulated the expression of *ntrB* in *A. citrulli*, which promoted the phosphorylation of NtrC to cope with nitrogen deficiency stress (results not shown). In summary, *ntrC* plays a key role in the nitrogen metabolism of *A. citrulli*.

The *A. citrulli ntrC* gene deletion mutant showed significantly heightened tolerance to oxidative, high salt, and copper ion-induced stress relative to Aac5, which is in agreement with previous reports [22,23]. In *Azospirillum brasilense*, phosphorylated NtrC is a transcriptional activator of the genes involved in nitrogen metabolism, which can promote the expression of the organic hydrogen peroxide resistance protein, Ohr [39]. Ohr belongs to the OsmC superfamily and has a detoxification effect on organic hydrogen peroxide [39]. In *Aeromonas hydrophila*, when *ntrC* was deleted, the expression levels of the NhaP type Na^+^/H^+^ and K^+^/H^+^ antiporter A0KFD8 were downregulated. This indicates that NtrC may use Na^+^/H^+^ and K^+^/H^+^ anti-transporters to cope with the stress of high osmotic pressure [40]. In *Escherichia coli*, the expression of RpoS, one of the RNA polymerase σ factors, was upregulated, and the expression of RpoS-dependent genes contributed to the universal resistance of cells [41].

*ntrC* has been deeply studied in nitrogen-fixing bacteria. In recent years, it has been reported that *ntrC* also plays an important role in the process of virulence in pathogenic bacteria. An *ntrC* mutant of *Pseudomonas aeruginosa* showed a reduced ability to invade and cause damage in a high-density abscess model in vivo [24]. In this study, *ntrC* deletion significantly impaired the virulence of *A. citrulli*. Virulence-related phenotypic assays showed that in vivo colonization, in vitro growth, swimming motility, and twitching motility of the *A. citrulli ntrC* mutant were significantly reduced, while the biofilm-forming ability was significantly enhanced. The weakening of swimming and twitching motility not only directly affected the ability of *A. citrulli* to infect plant tissue; it also affected the formation of biofilms [42,43]. The decrease in vivo colonization and in vitro growth may be related to the metabolic slowdown after the deletion of *ntrC*. Although the *ntrC A. citrulli* mutant entered the logarithmic growth phase 8 h later than Aac5 in vitro, the population of the mutant strain was higher than that of Aac5 after entering the stationary phase. This observation was consistent with the phenotype associated with the deletion of the flagellum-related gene *flgM* in *A. citrulli* [44]. In Aac5Δ*ntrC*, the expression of flagellum-related genes (*flhD*, *flhC*, *fliA*, and *fliC*) was significantly upregulated except for *filM*. Similarly, in the *A. citrulli flgM* deletion mutant, *flhD* and *fliC* genes were also significantly upregulated [44]. We speculate that flagellum-related genes may be closely related to the growth ability of *A. citrulli*. However, the swimming ability of the complemented strain was not restored to wild-type levels and was weaker than that of the *ntrC* mutant. These results were confirmed by the qRT-PCR assay. The expression levels of *flhD*, *flhC*, *fliA*, and *fliC* were not restored in the complemented strain, Aac5Δ*ntrC*comp, but were more upregulated than in Aac5Δ*ntrC* (results not shown). In order to further explain this observation, the flagella of Aac5, Aac5Δ*ntrC*, and Aac5Δ*ntrC*comp were observed, and their swarming motility was tested. We found that the deletion of *ntrC* did not affect the production of flagella nor the swarming motility of *A. citrulli* (results not shown). A recent study also found that swimming ability was completely lost after the deletion of a transcriptional regulatory factor, OxyR, in *A. citrulli*, and the complementary strain did not restore swimming ability [3]. One possibility is that the levels of NtrC may require precise regulation, possibly involving anti-sigma factors and anti-anti-sigma factors, for the proper function of flagellum-related genes [44,45,46]. In addition, the expression of ntrC was different between complementary and wild-type strains because the complementation is provided by a plasmid. The regulation of flagellum-related genes may require more precise regulation. Therefore, the regulatory mechanism of *ntrC* on swimming motility needs further investigation. In addition, the expression levels of the key *A. citrulli* T3SS genes *hrpE*, *hrpX*, and *hrcJ* were significantly downregulated in Aac5Δ*ntrC*, while the change in the expression of *hrpG* was not significant. However, it was reported that in *Xanthomonas oryzae* pv. *Oryzicola*, the expression of *hrpE* was not regulated by *hrpG* and *hrpX* [47], and the iron transport family regulator Zur could regulate the expression of *hrcC* and *hrpX*, but not *hrpG* [48]. Therefore, we speculate that *ntrC* is a potential regulator upstream of hrpE and hrpX.

In conclusion, *ntrC* not only plays an important role in nitrogen utilization and stress tolerance, but also contributes to the virulence of *A. citrulli*. Because of its important role in the regulation of the nitrogen metabolism, *ntrC* has been widely studied in nitrogen-fixing bacteria, but there are few studies on virulence in pathogenic bacteria. Future work should continue to search for the downstream targets of *ntrC* in *A. citrulli* and explore its regulatory network to identify new targets to improve the management of pathogenic bacteria such as *A. citrulli.*

## Data Availability

Not applicable.

## Supplementary Materials

The following supporting information can be downloaded at https://www.mdpi.com/article/10.3390/microorganisms11030767/s1. Table S1: Strains and plasmids used in this study. Table S2: Primers used for amplification of the target fragments.
