# Supplementary material for: ntrC Contributes to Nitrogen Utilization, Stress Tolerance, and Virulence in Acidovorax citrulli
Source: Microorganisms. 2023 Mar 16;11(3):767. doi: 10.3390/microorganisms11030767 (PMC10052978; doi:10.3390/microorganisms11030767)
Supplement: Supplementary file 1 [file microorganisms-11-00767-s001.zip › microorganisms-2276500-supplementary.pdf]

Supplementary Materials

Supplementary Materials: The following supporting information can be downloaded at: www.mdpi.com/xxx/s1, Table S1: Strains and plasmids used in this study; Table S2: Primers for amplification of target fragments.

Table S1. Strains and plasmids used in this study

Strain or plasmid	Description	Reference or source
Strain		
Escherichia coli DH5*α*	Φ80*lac*ZΔM15Δ (*lac*ZYA-argF) U169*end*A1*rec*A1 *hsd*R17*sup*E44 thi-1 *gyr*A96 *rel*A1 *pho*A	Tiangen, China
Aac5-WT	*Acidovorax citrulli*, wild-type, Amp^R	Lab collection
Aac5	Aac5-WT containing the empty vector pBBR1MCS-2, Amp^R, Km^R	This study
Aac5Δ*ntrC*	*ntrC* gene deletion mutant derived from Aac5, containing empty vector pBBR1MCS-2, Amp^R, Km^R	This study
Aac5Δ*ntrC*comp	Δ*ntrC* complemented with *ntrC* gene, Amp^R, Km^R	This study
Plasmid		
pK18*mobsacB*	Suicide vector for mutagenesis, Km^R	Lab collection
pK18-*ntrC*	Suicide vector carrying DNA fragment for generating mutant Δ*ntrC*, Km^R	This study
pBBR1MCS-2	Broad host expression vector, Km^R	Lab collection
pBBR1MCS-*ntrC*	Broad host expression vector carrying *ntrC* gene for Δ*ntrC*comp, Km^R	This study

Km^R and Amp^R indicate resistance to kanamycin and ampicillin, respectively.

Table S2. Primers for amplification of target fragments

Primers	Primer sequence (5′-3′)	Target length/bp
ntrC-1F	CTATGACATGATTACGAATTCGCCGAATACACCCAGGTCAT	592
ntrC-1R	AGCGGAACGGATGAGCAATCGAAGTGAATGTCTCCGGAC	
ntrC-2F	GTCCGGAGACATTCACTTCGATTGCTCATCCGTTCCGCT	532
ntrC-2R	ACGACGGCCAGTGCCAAGCTTTGCCTCGTCCCACTCCAT	
Δ*ntrC*comp-1F	GGCGGCCGCTCTAGAACTAGTGGCGATTGGGGGATACTTC	676
Δ*ntrC*comp-1R	AGGGAATGAGGATGCGGAAGCCGTCCTGATGCAGCA	
Δ*ntrC*comp-2F	TGCTGCATCAGGACGGCTTCCGCATCCTCATTCCCT	1763
Δ*ntrC*comp-2R	GGTACCGGGCCCCCCCTCGAGCGAAAACAGGAGCAAACCG	
Δ*ntrC*-F	CGGAGACATTCACTTCGAGAC	447
Δ*ntrC*-R	CGCTCCTCCGTGACCTCT	
WFB1	GACCAGCCACACTGGGAC	360
WFB2	CTGCCGCACTCCAGCGA	
Kan-F	CAAGATGGATTGCACGCA	738
Kan-R	CTTGAGCCTGGCGAACA	
ntrC-qF	GGCCTGCCGGTCATCATC	158
ntrC-qR	ACCTCTTCCCGCTGGCTTT	
nasS-F	TGAACGGCGAACTGGACTT	116
nasS-R	GCCGTTGTTGTTGAGGGTC	
nasT-F	TTCAACCTGGTGGCGACG	173

nasT-R	TCGTCGTTGGTGAACAGCA	
flhD-F	TTCGTTGCCGACCTTCTTG	171
flhD-R	TTCCGCCTGGGCATCAA	
flhC-F	TGGAACTGCTCACCAAGGC	126
flhC-R	GCGTGAGCATACCGGCAT	
fliA-F	ACACCGCCAAAGGACAGCT	178
fliA-R	CCCTGCGTCACCTCGTAGC	
fliC-F	GCTCGTTGACGATTTCCAT	179
fliC-R	TTCCTTTCCCAGGAAGAAGT	
fliM-F	ATCGTCTGCGAGCCGTCCCT	179
fliM-R	AGCGGGTAGATGCCGTGCC	
hrpG-F	CTCGCCTGGCTGCTGTT	197
hrpG-R	GCTTGTAGATGTGCTGCTCC	
hrpE-F	GTCAGGATGGACACGCAGGC	119
hrpE-R	AACGCATGGTGCTGGCAGAG	
hrpX-F	GCGCTCACGCAAATGCT	175
hrpX-R	GGCAAGCTCCTCCTGTCCTA	
hrcJ-F	CGGCAAGACCTGGAACG	174
hrcJ-R	GCGACGCCATAGATGAAGC	
pilA-F	TCAAGACGACTGCCGATGG	150
pilA-R	AAGAGCCAACTTGCTGCCC	
pilN-F	CTGGAGGGGCAGATCAAGG	175
pilN-R	CCGCCTGCTTCAGACTCG	
